# Temporal pulse shaping aspects of refractive X-ray lenses

**DOI:** 10.1107/S1600577525008975

**Published:** 2026-01-01

**Authors:** Fabian Trost, Johan Bielecki, Richard Bean

**Affiliations:** a European XFEL Holzkoppel 4 22869Schenefeld Germany; Australian Synchrotron, Australia

**Keywords:** X-ray optics, pulse streching, XFEL, CRL, attosecond science

## Abstract

Refractive X-ray lenses are frequently used components at modern X-ray free-electron laser facilities. This work investigates the temporal effects of refractive optics on ultra-short X-ray pulses, particularly focusing on pulse elongation. We present a model using full Fresnel theory to study the beam profile at the focus in space and time. Refractive X-ray lenses not only change the temporal structure of the pulse, the focus size is also dependent on the incoming pulse duration. Further, we present a simplified ray-tracing model to estimate the pulse stretching effect of compound refractive lenses (CRLs), which we find to be dependent only on the dispersion and the absorption of the CRL material.

## Abstract 2

This work investigates the temporal effects of refractive X-ray lenses on ultra-short pulses from X-ray free-electron lasers. Using both a full Fresnel theory model and a simplified ray-tracing approach, the paper analyzes the pulse elongation and spatio-temporal distortions introduced by these focusing optics.

## 1. Introduction

Modern X-ray free-electron lasers (XFELs) have revolutionized scientific research with X-rays by providing ultra-short, high-intensity X-ray pulses, enabling unprecedented insights into the fundamental dynamics of matter. These facilities can generate X-ray pulses with durations as short as a few hundred attoseconds, as demonstrated at the Linac Coherent Light Source (LCLS) (Huang *et al.*, 2017 ▸) and the European XFEL (Trebushinin *et al.*, 2023 ▸). Such ultra-short pulses have paved the way for groundbreaking experiments, including studies of nonlinear electron dynamics (Funke *et al.*, 2024 ▸), attosecond-scale processes in matter (Błachucki *et al.*, 2022 ▸), and innovative imaging techniques such as incoherent diffraction imaging (Trost *et al.*, 2023*a* ▸). These advances are critical for disciplines ranging from material science and chemistry to biology and quantum physics.

The focusing of such short pulses presents unique challenges. The finite speed of light becomes a significant factor in the attosecond regime, where the effects of optical components, such as X-ray lenses, on the temporal structure of the pulses cannot be ignored. Specifically, refractive optics, which are commonly employed to focus X-ray beams, introduce temporal distortions due to their dispersive properties (Chapman & Bajt, 2021 ▸). These distortions are caused by variations in path length and group velocity within the lens material.

This study investigates the temporal pulse shaping effects of refractive X-ray lenses, especially compound refractive lenses (CRLs) (Tomie, 2010 ▸; Snigirev *et al.*, 1996 ▸). CRLs, composed of multiple lens elements, are widely used in XFEL applications due to their ability to focus high-energy X-rays. Using theoretical models based on Fresnel optics and geometrical ray-tracing, we examine the propagation of Gaussian pulses through these lenses.

## 2. Propagation through a refractive lens in Fresnel theory

In this section we derive the propagation of a light pulse through a refractive lens utilizing Fresnel theory. For our analysis, we assume a spatially and temporally Gaussian shaped wave-packet, given by 
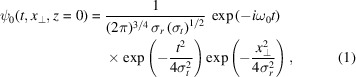
where ω_0_ denotes the central (angular) frequency of the carrier wave, σ_*r*_ the spatial and σ_*t*_ the temporal standard deviation of the Gaussian shape. The wave packet is normalized such that the intensity^1^*I*(*t*, *x*_⊥_, *z*) = ψ^*^ψ integrated over time and the plane orthogonal to the optical axis is unity: 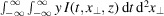 = 1. Here, 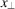 := 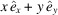 represents the component of the spatial vector orthogonal to the optical axis 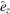. The standard deviation (SD) in the orthogonal plane and in time are given by σ_*r*_ and σ_*t*_, respectively. We make use of the frequency representation for the temporal pulse shape, given by 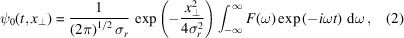
with the spectrum 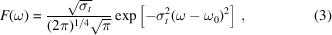
where the central frequency ω_0_ is determined by the photon energy *E* via the relation ω_0_ = *E*/ℏ.

**Figure 1:**
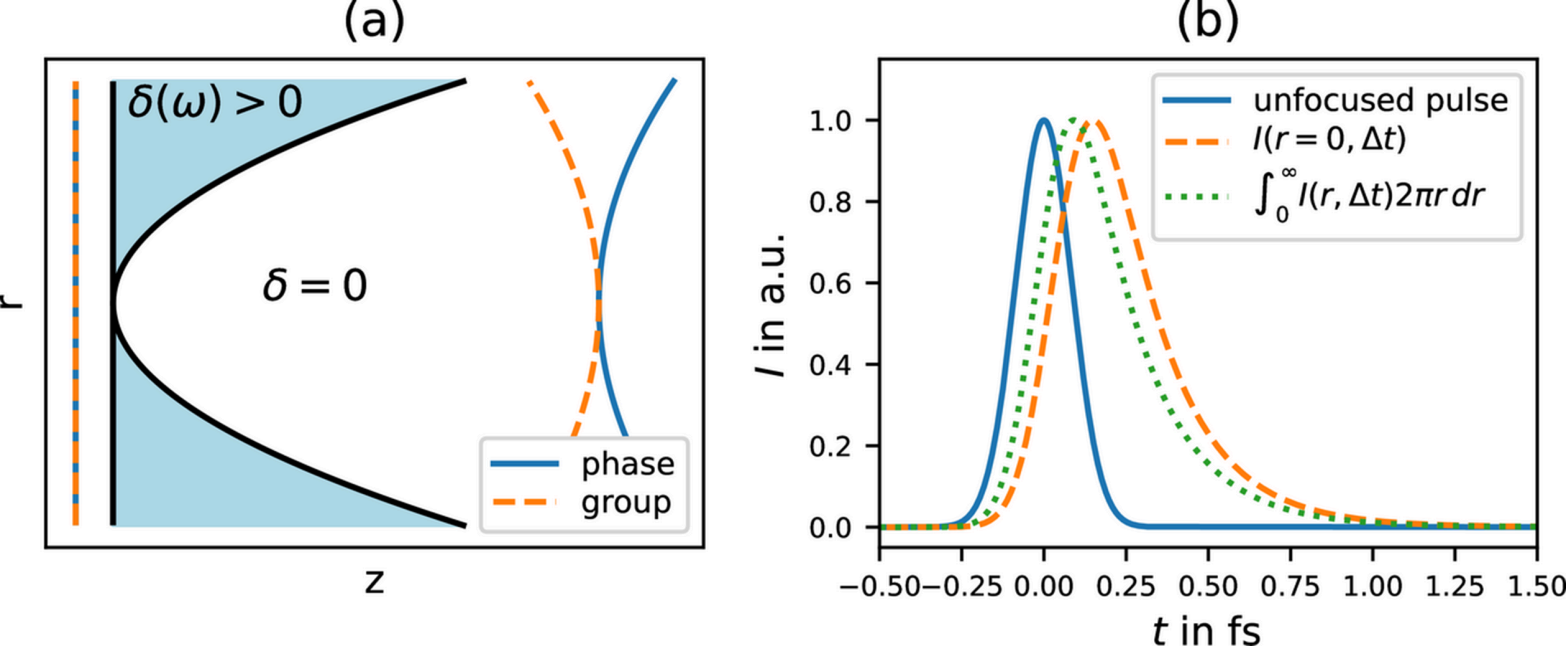
(*a*) Sketch of an ideal paraboloid X-ray lens. Refractive index *n* = 1 − δ < 1, such that the phase velocity in the lens *c*_ph_ > *c*_0_, while the group velocity *c*_gr_ < *c*_0_. (*b*) Temporal intensity profile *I*(*t*) evaluated in the focal plane. Unfocused X-ray pulse (blue, solid), focused pulse at *r* = 0 (orange, dashed) and spatially integrated over the focus plane (green, dotted). The displayed pulses were individually scaled such that their maximum is unity. The incoming pulse has a duration of 200 as FWHM, a size of 755 µm FWHM and mean photon energy *E*_0_ = 12 keV. The assumed Be lens has a focal length of *f* = 3 m. Note that the pulse at *r* = 0 is more delayed and wider (345 as FWHM) than the spatially integrated one (318 as FWHM).

To model the X-ray lens, we assume a perfect paraboloid lens with infinite extent, and zero thickness at the center (*r*_⊥_ = 0), as illustrated in Fig. 1 ▸(*a*). In addition, we make use of the projection approximation (Paganin, 2006 ▸), which collapses the extent of the lens along the optical axis to 0. Now the only effect of the lens is to introduce a phase shift at *z* = 0, 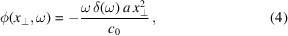
where *a* denotes the parabola factor^2^ and δ(ω) is the deviation of the refractive index from unity: *n*(ω) = 1 − δ(ω). Typically for X-rays, 0 < 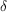

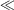
 1, such that the phase velocity exceeds the vacuum speed of light (*c*_ph_ > *c*_0_) inside the material. At this point we neglect the absorption inside the lens material but will return to it later.

As we are interested in monochromatic beams with a relatively narrow spectrum (of the order of 1 eV), we can expand the refractive index around the central frequency ω_0_ to first order, 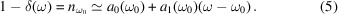
For most of the example calculations within this paper, we choose a photon energy of 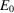 = 12 keV 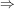

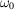
 = 1.823 × 10^19^ s^−1^ and assume lenses made of beryllium. In that case, the parameters are *a*_0_ = 1 − 2.366 × 10^−6^ and *a*_1_ = 2.590 × 10^−25^ s (Henke *et al.*, 1993 ▸).The resulting focal length of such a lens is given by 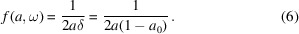
Following these preparations, we can now add the phases applied by the lens from equation (4) to the initial wavefield as given in equation (2) and propagate to the focal point. The propagation is completed by convoluting each frequency component with the Fresnel propagator
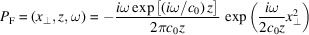
(Goodman, 1996 ▸). We obtain 
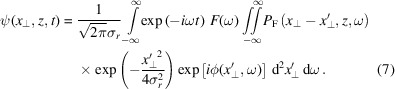
Since there is no closed-form solution of this integral (only partial solutions), we need to evaluate it numerically. Details about the evaluation and its numerical convergence as well as an argument why there is no closed-form solution can be found in Appendix *A*[App appa]. A similar calculation is given by Gu (2000 ▸) for visible light. However, all the effects we discuss in the following are more significant in the X-ray regime due to the refraction indices being smaller than unity and the waves phase- and group-front are shifted in opposite directions, as illustrated in Fig. 1 ▸(*a*).

To investigate the pulse shaping effects on the X-ray pulse by the lens, we first assume an incoming pulse with a flat (top hat) spatial intensity distribution and Gaussian temporal profile with full width at half maximum (FWHM) = 200 as (σ_*t*_ = 84.9 as), which corresponds to the duration of a typical single self-amplified spontaneous emission (SASE) spike (Huang *et al.*, 2017 ▸). For the Be X-ray lens, we assume a focal length of *f* = 3 m which implies a parabola factor of *a* = 70447 m^−1^. Absorption inside the lens is calculated using the projection approximation. Applying the Bouguer–Lambert–Beer law (Bouguer, 1729 ▸), we obtain the radial intensity distribution downstream of the lens as 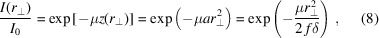
with the absorption coefficient for Be μ_Be_(12 keV) = 69.03 m^−1^ (Henke *et al.*, 1993 ▸). The choice of an incoming pulse with flat intensity distribution is an interesting case to study since it can be interpreted as the limit of maximally achievable numerical aperture for most realistic setups. Absorption inside the lens shapes the spatial profile of such a pulse into a Gaussian radial profile with 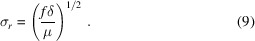
Note that the relation 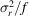 = 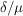 is fully determined by the lens material constants δ and μ, which turns out to be important as we will discuss later in this work (in Section 3). For our example setup we obtain σ_*r*_ = 320.7 µm.

In Fig. 1 ▸(*b*) the temporal profile of the pulse is plotted in the focal plane for an unfocused beam without lens (solid blue), for the focused beam at the position 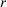 = 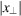 = 0 (orange dashed) and focused beam integrated over the focal plane orthogonal to the optical axis 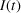 = 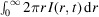 (green dotted). We observe that the pulse at the lens focus is stretched compared with the incoming pulse. Further, the pulse measured at *r* = 0 is more delayed, and longer (345 as FWHM) in comparison with the integrated one (318 as FWHM). The stronger stretching and delay closer to the optical axis (smaller *r*) is caused by a larger phase shift at higher numerical aperture (NA) due to the parabolic shape of the lens.

**Figure 2:**
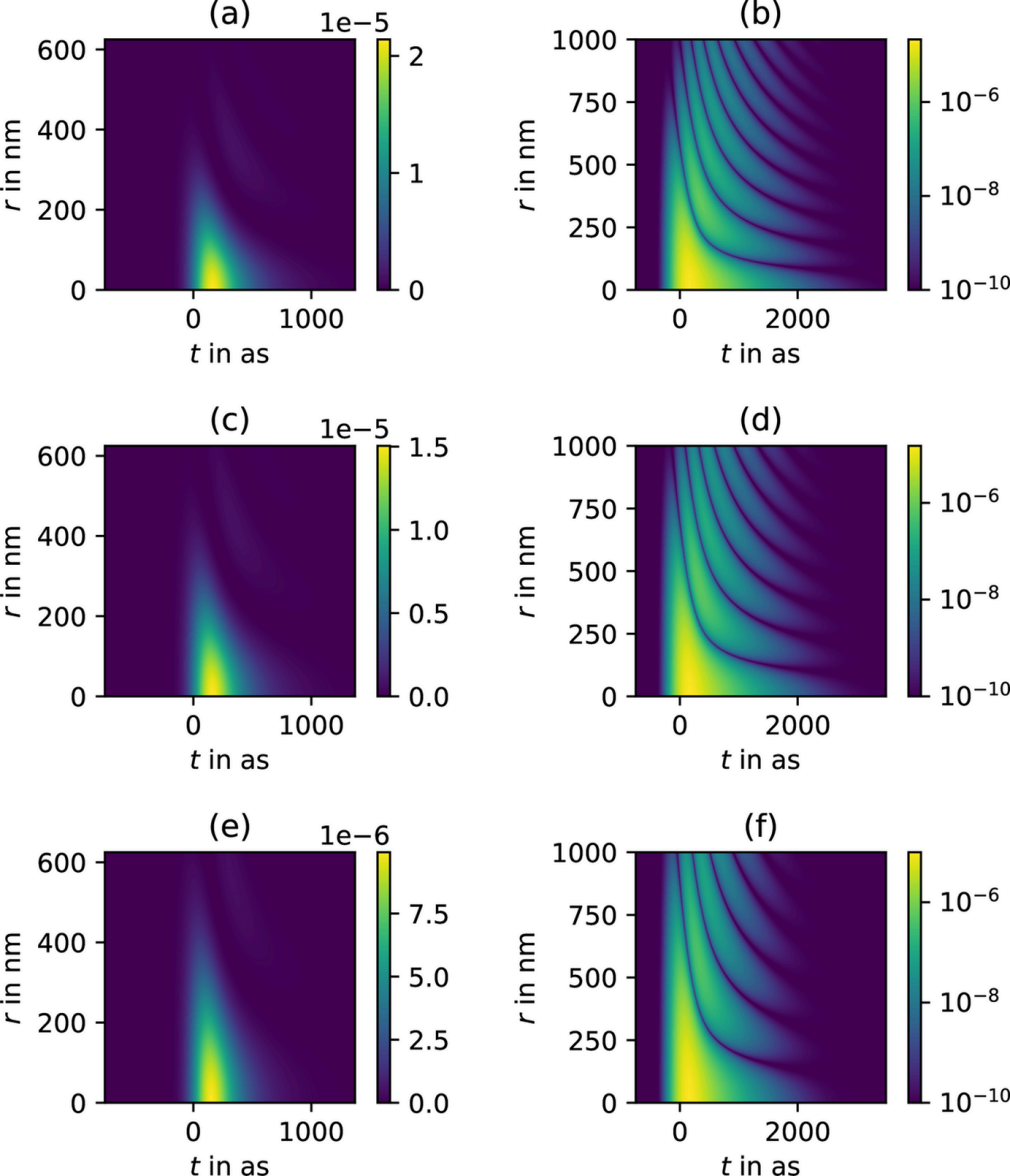
*I*(*x* = *r*, *y* = 0, *z* = *f*, *t*) = ψ^*^ψ from equation (7) evaluated at the focus for an initial 200 as Gaussian pulse with *f* = 3 m. (*a*, *b*) *E*_0_ = 12 keV and σ_*r*_ = 321 µm; (*c*, *d*) *E*_0_ = 10 keV and σ_*r*_ = 313 µm; (*e*, *f*) *E*_0_ = 8 keV and σ_*r*_ = 290 µm. The values in (*b*), (*d*) and (*f*) are elevated by the summand 10^−10^ to enable a good logarithmic representation. Note that the pulse stretching effect gets weaker for smaller σ_*r*_.

For a closer investigation, the intensity at the focal plane is plotted as a function of time, and the radial distance from the optical axis in Fig. 2 ▸ for three different photon energies *E* = 12 keV, 10 keV and 8 keV, maintaining the focal length but adjusting the beam size accordingly to equation (8). Note that these plots represent a cut along *r*, without polar integration. As expected, the focus becomes smaller at later times since the higher NA parts are more delayed. Consequently, the pulse gets shorter the further away we get from the optical axis. The logarithmic representation in Figs. 2 ▸(*b*), 2(*d*) and 2(*f*) also reveals an oscillatory behavior.

### 2.1. Focus size

As mentioned in the previous section, photons arriving later produce a smaller focal spot size, since the inner part of the beam (smaller NA) is less delayed than the outer part (higher NA). For a sufficiently long initial pulse this effect can safely be neglected, since for the majority of the contained photons the beam is focused with a constant NA.

**Figure 3:**
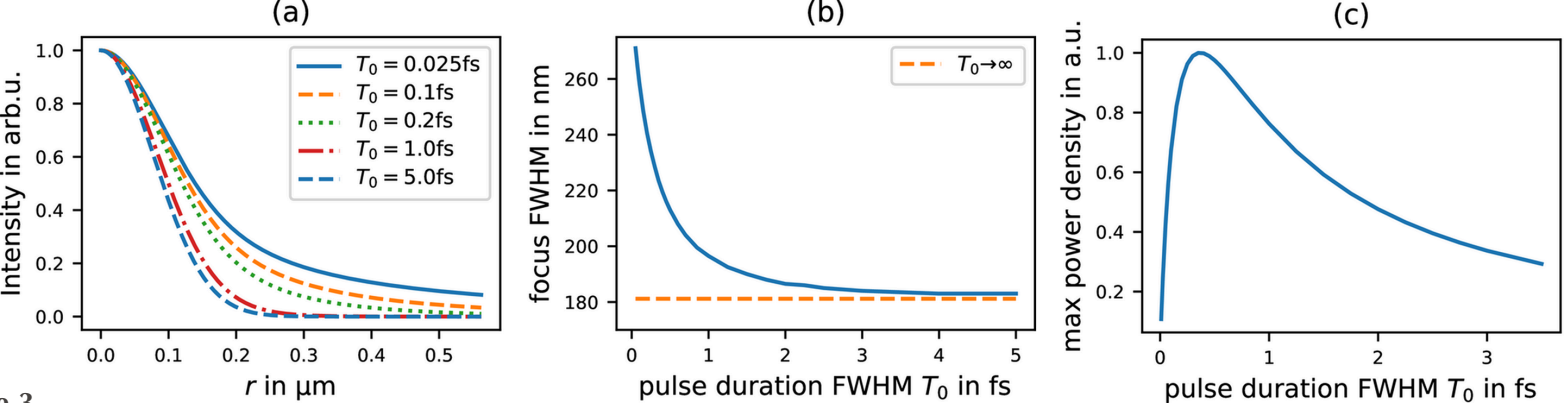
Focus size dependence on incoming pulse duration (*T*_0_). Initial Gaussian beam with 755 µm FWHM, 12 keV photon energy propagating through a Be lens with *f* = 3 m. (*a*) Radial cut of time integrated intensity at the focus for different *T*_0_. (*b*) Spatial FWHM of the beam at the focus as a function of the incoming pulse duration *T*_0_. The limit for infinite long pulses is 181.2 nm FWHM. (*c*) Maximum power density at the focus. Peak at *T*_0_ ≃ 350 as initial pulse duration.

To demonstrate this effect, we assume the same incoming X-ray pulse with a photon energy of 12 keV (Gaussian shape, with spatial FWHM = 755 µm) and the duration *T*_0_ (FWHM). Again, the focusing lens is assumed to be made of Be material with a focal length of 3 m at the given photon energy. In Fig. 3 ▸(*a*) the radial profile of the pulse at the focus is plotted for different pulse durations *T*_0_. We can see a clear dependence of the focus size, which saturates to the diffraction limit 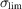 = 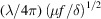 for longer pulses, as displayed in Fig. 3 ▸(*b*), where the beam width (FWHM) at the focus is plotted as a function of *T*_0_.

We also like to highlight that the relation between focus size and pulse duration implies, given constant number of photons, the existence of an optimal pulse duration in terms of power density. In Fig. 3 ▸(*c*) the maximal power density is plotted as a function of pulse duration where we find the maximum at *T*_0_ = 350 as.

## 3. Propagation through a lens using ray-tracing

In this section, we will compare the previously obtained full Fresnel-theory results with a simple ray-tracing model (geometrical optics). The main result in this section is an analytical approximation of the pulse stretching kernel. In addition we show the validity of the thin lens approximation employed in the Fresnel propagation in the previous section.

The temporal pulse shape at the focus is obtained via convolution of the incoming pulse with the impulse response function *I*_δ_(*t*), which describes the temporal response of a δ-function pulse and acts as a stretching kernel,
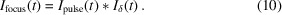
*I*_δ_(*t*) is obtained by the time delay Δ*t* caused by a combination of the group velocity inside the lens and the geometrical path length differences for each ray in the beam.

Assuming a Gaussian spatial beam profile and cylindrical symmetry, the problem depends only on the radial coordinate *r*_⊥_ and the impulse response function can be written as 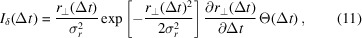
where θ(Δ*t*) denotes the Heaviside step function^3^. The factor ∂*r*_⊥_(Δ*t*)/∂Δ*t* is required for normalization of the intensity distribution to be maintained when integrating over Δ*t*.

Next we find *r*_⊥_ as a function of Δ*t*. The path length of a ray from the lens plane (still assuming a thin lens) to the focus is 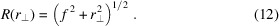
We can express the time delay Δ*t* of a ray at an initial distance *r*_⊥_ to the optical axis as 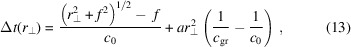
where *c*_gr_ is the group velocity inside the lens material.

**Figure 4:**
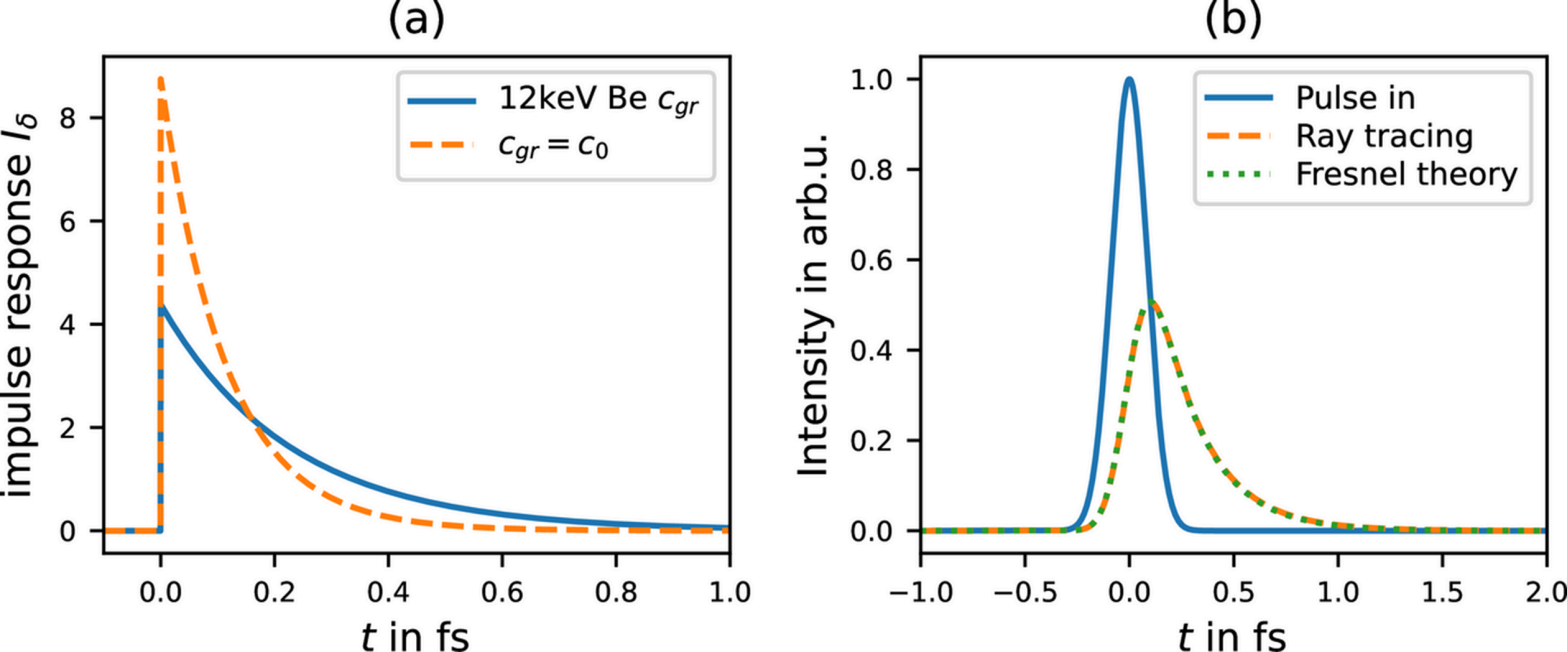
(*a*) Impulse response function *I*_δ_ for a Be lens with *f* = 3 m, a beam size 755 µm FWHM, and a photon energy of 12 keV. The solid blue line considers the corresponding group velocity inside the lenses material; the dashed orange line neglects the reduced group velocity. (*b*) Pulse-stretching effect of a 200 as FWHM Gaussian pulse. For the Fresnel theory plot the intensity is spatially integrated. Note that the ray-tracing approach yields a result extremely similar to the one using the Fresnel theory.

We solve equation (13) for *r*_⊥_,
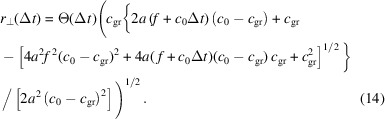
By substituting this result into equation (11) we obtain the delta-response function as 
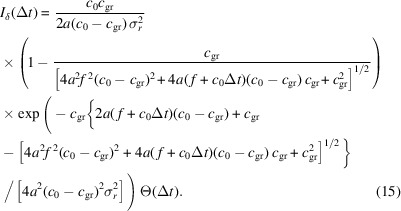
The impulse-response function for a Be lens with *f* = 3 m and 12 keV is plotted in Fig. 4 ▸(*a*), and its convolution with a Gaussian pulse of 200 as FWHM is plotted in Fig. 4 ▸(*b*). The comparison with the, also in Fig. 4 ▸(*b*), plotted result using the Fresnel theory shows a very good agreement. In this particular example, the difference between the ray-tracing and the Fresnel theory approach does at no point exceed 0.81% of the maximum value calculated using Fresnel theory.

### 3.1. Approximation

Since equation (15) cannot necessarily be assumed convenient, we want to bring it in a simpler, albeit approximated, form. In a first step we look at the limit where the group velocity in the lens matches the vacuum speed of light, 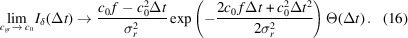
This is done purely for mathematical simplicity, since there is no physical realization of this case and thus we will drop that assumption later on.

Since we are interested in short pulses only, 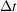

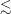
10^−15^ s 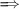

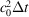

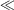

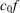
 and 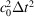

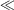

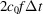
. Thus we can drop these much smaller contributions and obtain the following approximation,
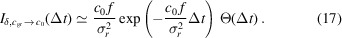
With the definition of the (modified) Fresnel number^4^, 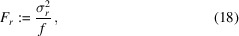
equation (17) can be written as 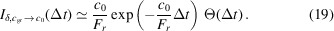
This is an elegant and compact form of the delta response function in a reasonable approximation. However, it does not match reality since usually *c*_gr_ < *c*_0_. But, we can adjust the (modified) Fresnel number such that the group velocity is taken into account. The *effective Fresnel* number then reads 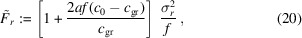
which requires, besides the group velocity *c*_gr_, also the parabola constant *a* of the lens. A derivation of equation (20) can be found in Appendix *B*[App appb]. With this, equation (19) simply becomes 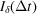

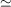

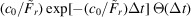
.

**Figure 5:**
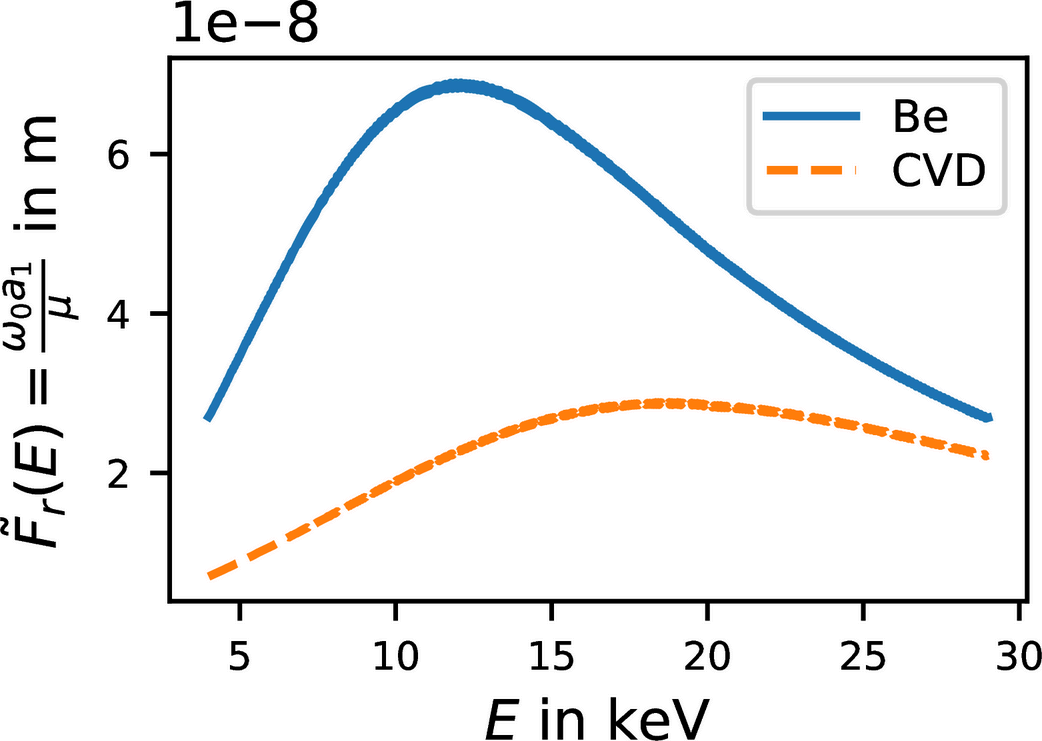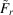 as derived in equation (21) plotted as a function of photon energy for beryllium (Be) and chemical vapor deposition diamond (CVD). The maxima values are 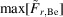 = 6.895 × 10^−8^ m at 12.089 keV for Be, and 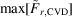 = 2.889 × 10^−8^ m at 18.777 keV for CVD, respectively. Data based on Henke *et al.* (1993 ▸).

In the previous section, we have discussed that due to absorption a flat incoming beam will become spatially Gaussian shaped by absorption. Substituting the relation of equation (8) into equation (18) or equation (20), and using the relations *f* = (2*a*δ)^−1^ and *c*_gr_ = *c*_0_/(*a*_0_ + ω_0_*a*_1_), we obtain 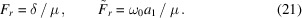
This 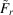 represents an upper limit of the pulse stretching since the assumption is basically an incoming flat beam of infinite extent yielding the maximum effective Fresnel number for a given lens, as expressed by equation (8). It is worth noting that 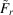 in the large, flat beam limit is only dependent on the photon energy (ω_0_), the dispersion (represented by *a*_1_) and the absorption, and therefore independent of the focal length. 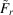 is shown as a function of photon energy in Fig. 5 ▸ for beryllium as well as CVD diamond. The maxima of 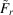 indicates that maximum time stretching occurs at a photon energy of 12.1 keV for Be and 18.8 keV for CVD lenses.

A beam with a spatial Gaussian profile before passing through the lens will result in a smaller 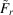 and thus less time stretching, namely 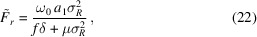
where σ_*R*_ denotes the spatial size (SD) of the incoming Gaussian beam. Be aware that both equation (21) and equation (22) are assuming an infinitely extended lens in the perpendicular direction to the optical axis and thus need to be adjusted when the size of the beam is restricted (*e.g.* by a pinhole or a finite sized lens).

### 3.2. Compound refractive X-ray lens

The good agreement of the stretched pulse calculated with ray-tracing and Fresnel theory justifies the use of ray-tracing. In the next step, we drop the assumption of a thin lens and assume a more realistic setup.

**Figure 6:**
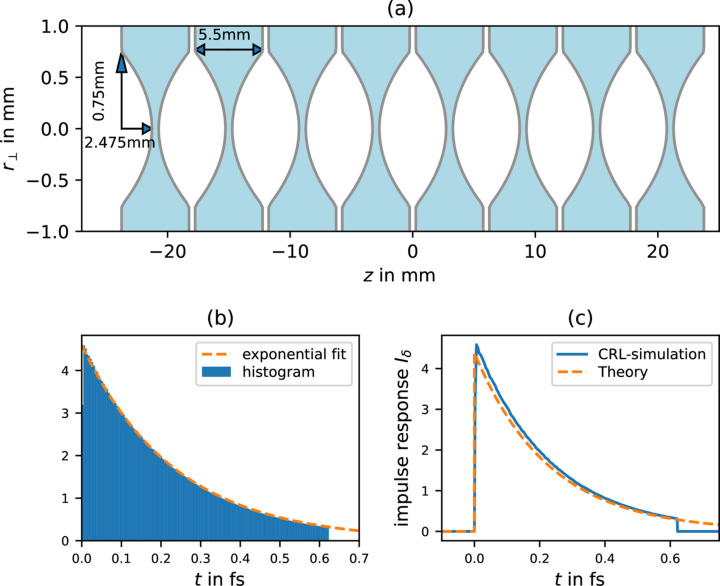
(*a*) Sketch of the simulated CRL consisting of eight Be X-ray lenses, with *f* = 3.01 m for 12 keV collimated rays. (*b*) Intensity-weighted histogram of time delays of the rays at the focus relative to the ‘fastest’ ray. The exponential fit yields 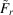 = (7.035 ± 0.073) × 10^−8^ m. (*c*) Impulse response function for the CRL simulation and the theory according to equation (15). The discrepancy is mainly caused by the cutoff due to the hard aperture cutoff in the simulation.

Therefore, we split the X-ray lens into eight symmetric double-sided paraboloid lenses (equal to 16 lenses) with an apex curvature radius of *R* = 113.636 µm on each side corresponding to a parabola factor *a* = 1/(2*R*) = 4400 m^−1^. A sketch of the resulting CRL is given in Fig. 6 ▸(*a*). For the simulation, a ray-tracing of 10^7^ rays that were randomly distributed within a 750 µm radius disk was performed. These initial rays are set to be perfectly collimated. The ray-tracing was performed using Snell’s law (Born & Wolf, 1999 ▸) at each Be–vacuum intersection. Furthermore, absorption within the Be material was considered as well as the reduced group velocity. The time each ray took from the circular flat source upstream of the CRL to the focus was calculated. Since we are only interested in the time delay between different rays the actual position of the source is of no interest. The focal length of this CRL (from the middle of the CRL to the focus) turned out to be *f* ≃ 3010.05 mm and thus quite similar to the 3 m we were using throughout this paper.

The relative time delay of each ray to the ‘fastest’ ray is plotted as an intensity-weighted histogram in Fig. 6 ▸(*b*). A fitted exponential function returns 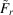 = (7.035 ± 0.073) × 10^−8^ m, which is reasonably close to the value calculated via equation (21): 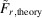 = 6.84 × 10^−8^ m for the ‘collapsed’ lens.

The resulting impulse-response function is plotted in Fig. 6 ▸(*c*) along with the theoretical one using equation (15). The cutoff at 0.62 fs for the CRL simulation is due to the pinhole-like finite initial beam extent (and the finite extent of the X-ray lenses). Considering the rescaling due to this cutoff (the impulse-response function must be normalized), the theory curve, that assumed a flat lens, agrees quite well with the simulated one.

## 4. Multi SASE-spike pulses

**Figure 7:**
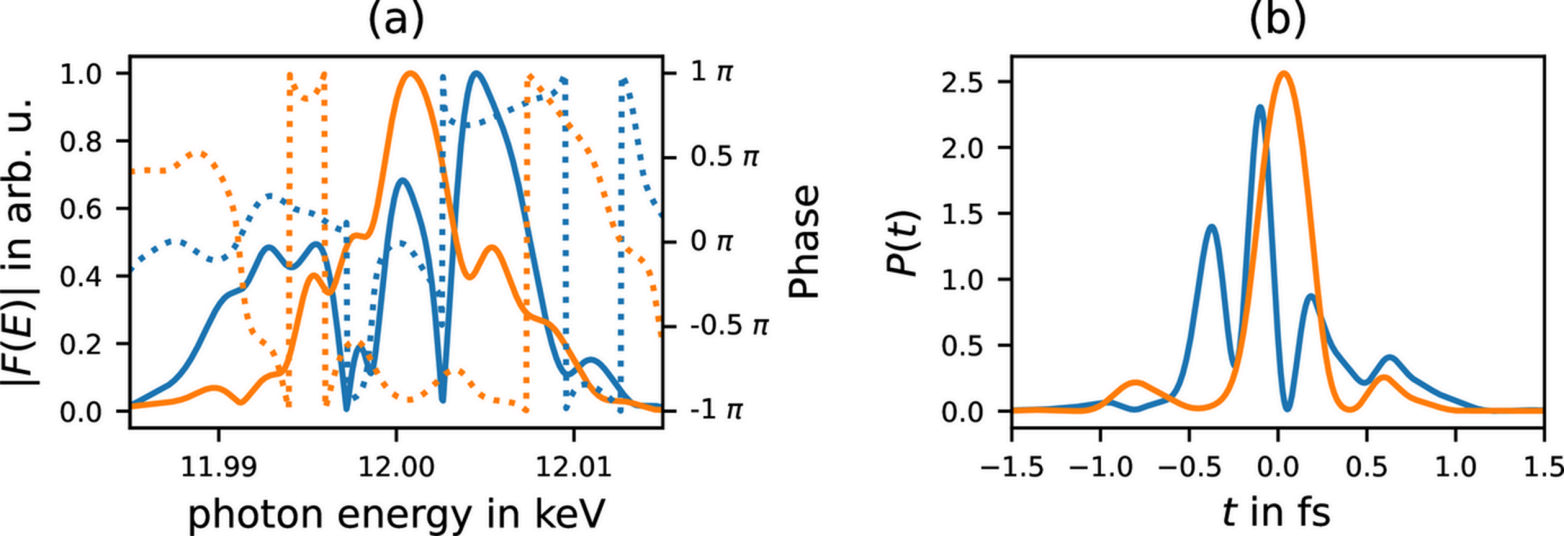
Two example SASE pulses. (*a*) Spectrum, with amplitudes (solid) and phase (dotted). (*b*) Corresponding temporal pulse shapes.

So far, we have assumed an idealized incoming Gaussian-shaped pulse, similar to a single, isolated SASE spike. In this section, we have a look at pulses consisting of multiple SASE spikes, as are usually produced by modern XFELs (Bonifacio *et al.*, 1994 ▸). Therefore, we have simulated a bunch of pulses (10 000 in total) using the heuristic method described by Pfeifer *et al.* (2010 ▸). A mean photon energy of 12 keV, and bandwidth of 0.1%, as well as a duration of the *averaged pulse*^5^ of 1 fs FWHM was assumed. Two exemplary spectra are plotted in Fig. 7 ▸(*a*) together with the resulting temporal pulse shapes in Fig. 7 ▸(*b*). Note that the average over many pulses is in general longer than each individual pulse, due to the jitter of the single SASE-spike positions. To evaluate the pulse at the focus we make use of the full Fresnel propagation as described in Section 2. Therefore, the spectra *F*(ω) were substituted into equation (7).

Since SASE pulses usually consist of multiple spikes, the FWHM becomes an unreliable measure for the length of a pulse. Thus, we propose two alternative measures for the width of a 1D curve, namely the minimum width of half area (MWHA) and the maximum value normalized integral (MVNI). The definition and a discussion about their properties is given in Appendix *C*[App appc].

**Figure 8:**
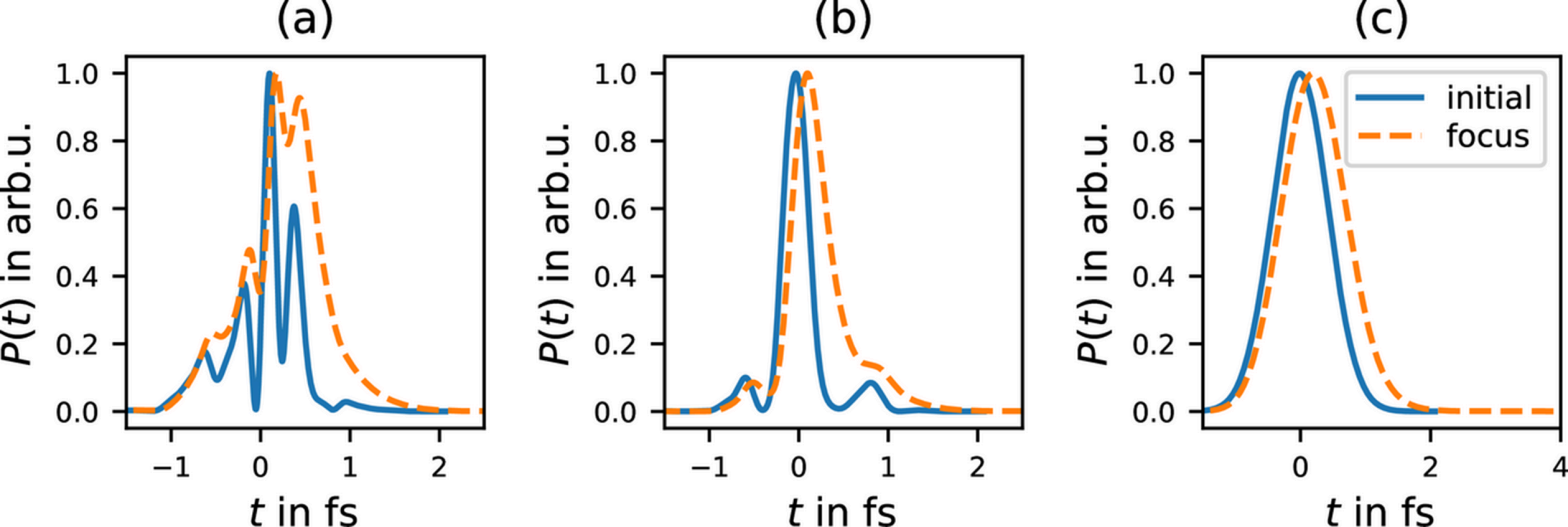
SASE pulses (12 keV, average 1 fs FWHM) before the lens (solid) and at the focus (dashed, focal length *f* = 3 m). (*a*, *b*) Two random examples. (*c*) Average of 10 000 SASE pulses.

Figs. 8 ▸(*a*) and 8(*b*) display two random examples of initial SASE pulses before the lens (solid lines) and at the focus (dashed lines). In this section we restrict our analysis on the spatially integrated intensity at the focus *I*(*t*) = 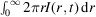.

The average over all 10 000 simulated pulses is shown in Fig. 8 ▸(*c*). The corresponding pulse-length metrics (FWHM, MWHA and MVNI) are given in Table 1 ▸. Here two different kinds of averages were used: first, the ‘width-functional’ (FWHM[*f*], MWHA[*f*] and MVNI[*f*]) was applied on the average over all 10 000 examples [*f* = 〈*I*(*t*)〉] – duration of the averaged pulse; and, second, the averaging was done after applying the width functional to each sample individually {*e.g.* 〈FWHM[*I*(*t*)]〉} – averaged pulse duration. We observe that the second case yields in general significantly shorter pulse length, which is expected since it is not sensitive to the pulse-to-pulse jitter. However, we also observe that the stretching of the pulses by the lens (see the ratio focus/initial) appears stronger when averaging the width values compared with the width of the averaged intensity.

**Figure 9:**
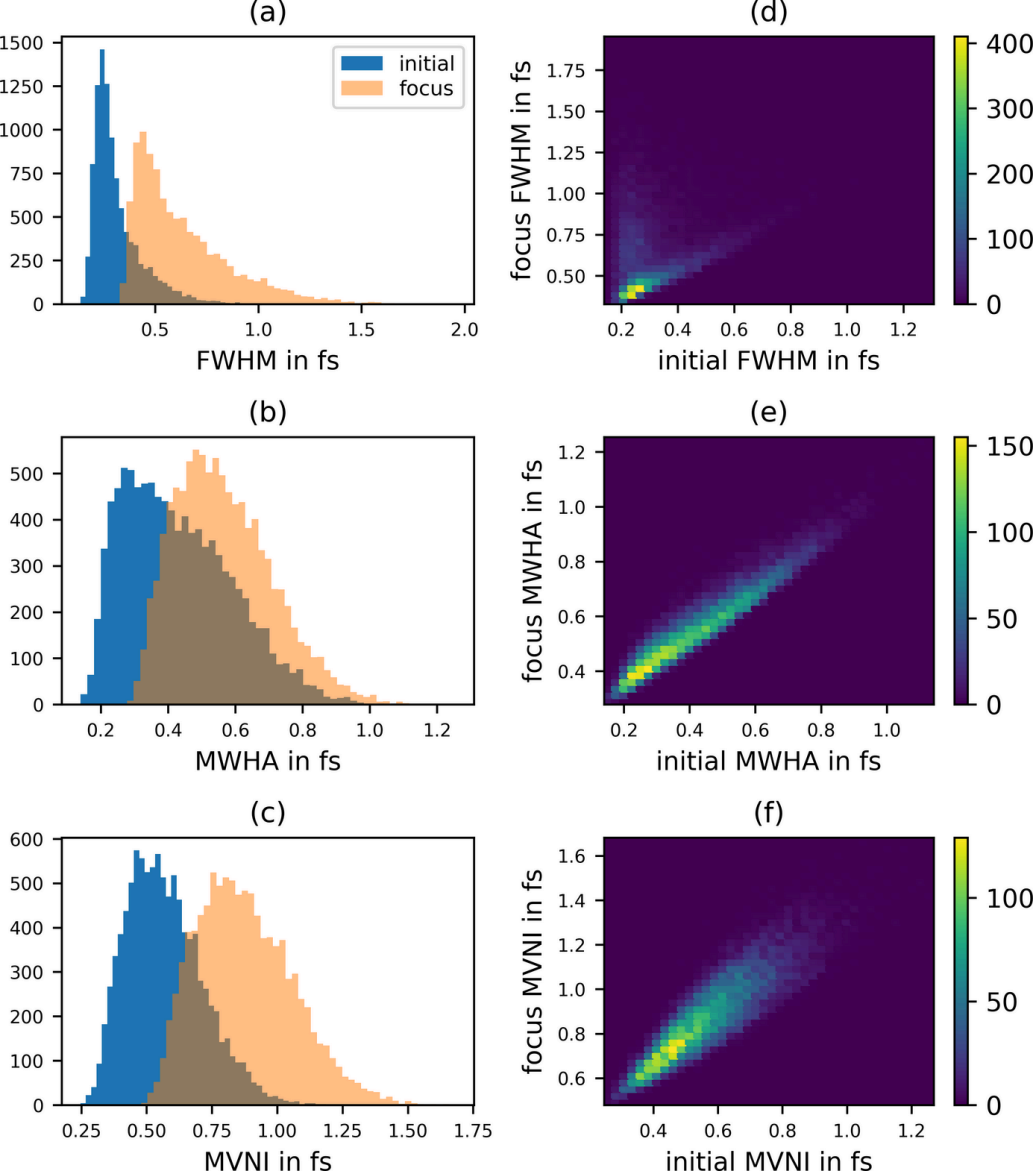
Statistics of the pulse length of 10 000 random SASE pulses with an average FWHM of 1 fs. Initial pulse before the lens, and pulse at the focus (*f* = 3 m). (*a*) Full width at half-maximum, (*b*) minimum width half area, (*c*) maximum normalized integrated value. (*d*, *e*, *f*) 2D histograms of the pulse width before the lens and at the focus.

Histograms of the three pulse-length metrics are displayed in Fig. 9 ▸, where the pulse stretching is clearly visible, and also the need of additional measures beside the FWHM is apparent.

**Figure 10:**
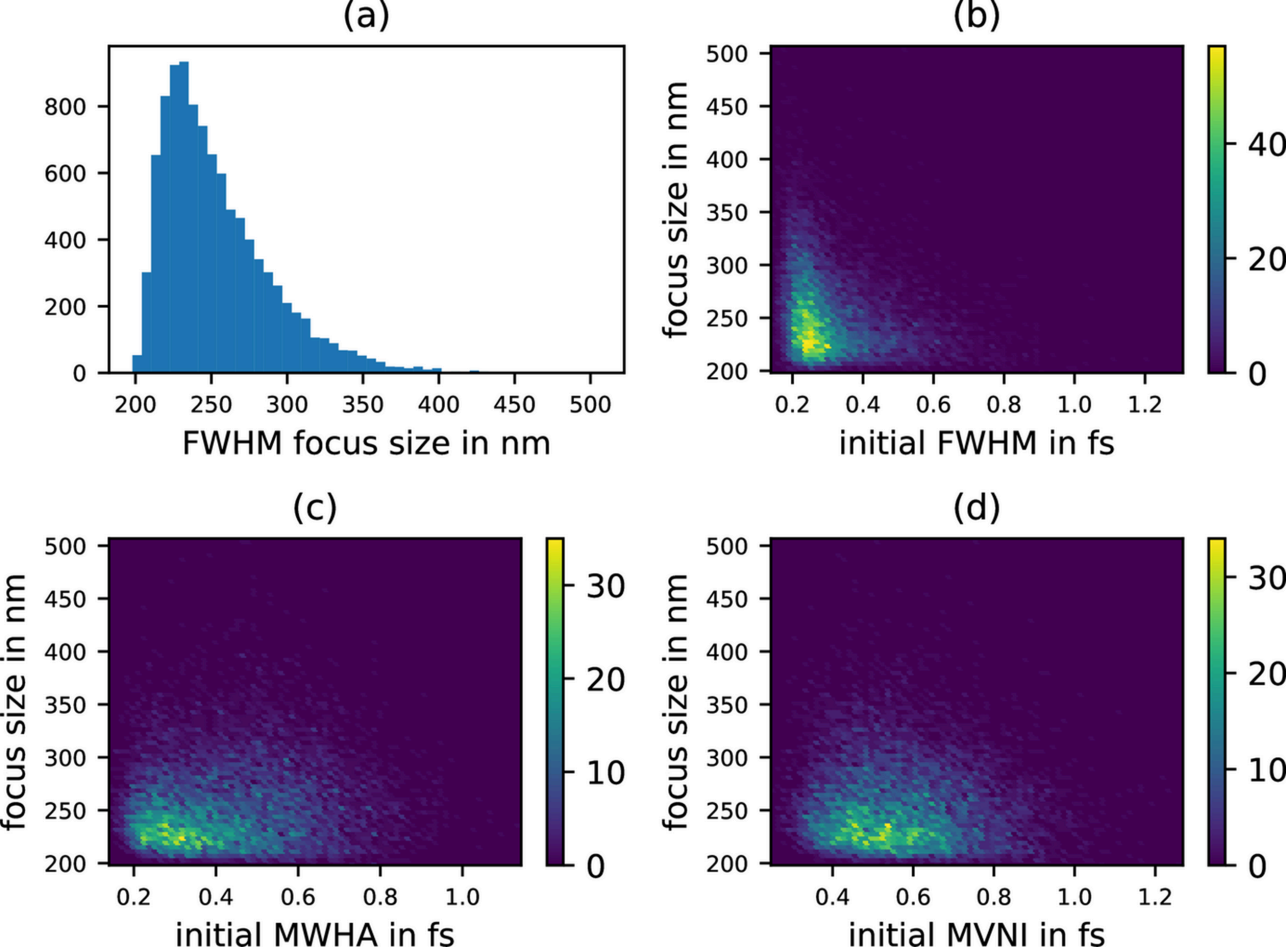
(*a*) Histogram of the spatial focus size of the 10 000 simulated SASE pulses. The largest focus is 507 nm FWHM. (*b*) Spatial focus size as a function of initial pulse duration in FWHM. (*c*) Spatial focus size as a function of initial pulse duration in MWHA. (*d*) Spatial focus size as a function of initial pulse duration in MVNI.

As discussed before, also the focus size changes with different pulse durations. A histogram of the spatial FWHM of the pulses at the focus is plotted in Fig. 10 ▸(*a*), showing a quite long-tailed distribution, where the minimum focus size is 198 nm FWHM and the maximum is 507 nm FWHM. In contrast to the Gaussian single spike case [recall Fig. 3 ▸(*b*)], where we were able to show a clear, smooth and continuous relation between the initial pulse duration and the spatial focus size, it is not really possible here, as shown in Figs. 10 ▸(*b*), 10(*c*) and 10(*d*). One could argue that short and spiky pulses lead to a higher probability that the spatial focus gets worse, as indicated by the temporal FWHM measure; however, there are also quite many good foci at short pulses in all three metrics.

## 5. Discussion

In this paper we investigated the spatial and temporal pulse shaping effects of refractive X-ray lenses on ultra-short pulses using both full Fresnel theory and geometrical ray tracing.

We quantified the amount of pulse stretching experienced by a Gaussian pulse, and found good agreement between the two approaches. In particular, we defined a modified Fresnel number that captures the degree of stretching as a function of lens material and photon energy. Be and CVD, two of the most common X-ray lens materials, exhibit maximal pulse stretching at 12.1 and 18.8 keV, respectively, a finding that does not depend on the CRL focal distance. For beams with uniform (flat) pre-lens intensity profiles, which acquire a Gaussian distribution solely through absorption within the lens, the pulse stretching depends only on material properties and photon wavelength, and in this case becomes independent of focal length.

Spatial pulse shaping was also observed, with shorter pulses focusing significantly worse than the classical diffraction limit. As a consequence of the interplay between spatial and temporal pulse shaping the instantaneous power density could be shown to have a maximum for 350 as pulses when using Be lenses.

Longer X-ray pulses, involving a larger number of SASE spikes, were also considered. In this case the pulse stretching was more complicated to describe due to interference between the different SASE spikes. As a consequence, different incoming pulses with nominally identical duration give rise to a wide distribution of focal spot sizes and effective pulse durations in the focus.

These findings show the importance of taking the focusing optics into account when planning experiments on non-linear X-ray matter interactions, a field that is currently emerging with the availability of sub-fs X-ray pulses at free-electron lasers. This is especially true for multi-spike SASE pulses with single fs duration, as the pulse-to-pulse variations in focal spot size and pulse stretching will make the interpretation of experimental results challenging. For such experiments, optics free from pulse stretching effects, such as Kirkpatrick–Baez mirrors, are likely the preferred choice. In cases where short pulse duration is prioritized over peak power density, restricting the spatial acceptance of the CRL may be a viable approach.

## Figures and Tables

**Figure 11:**
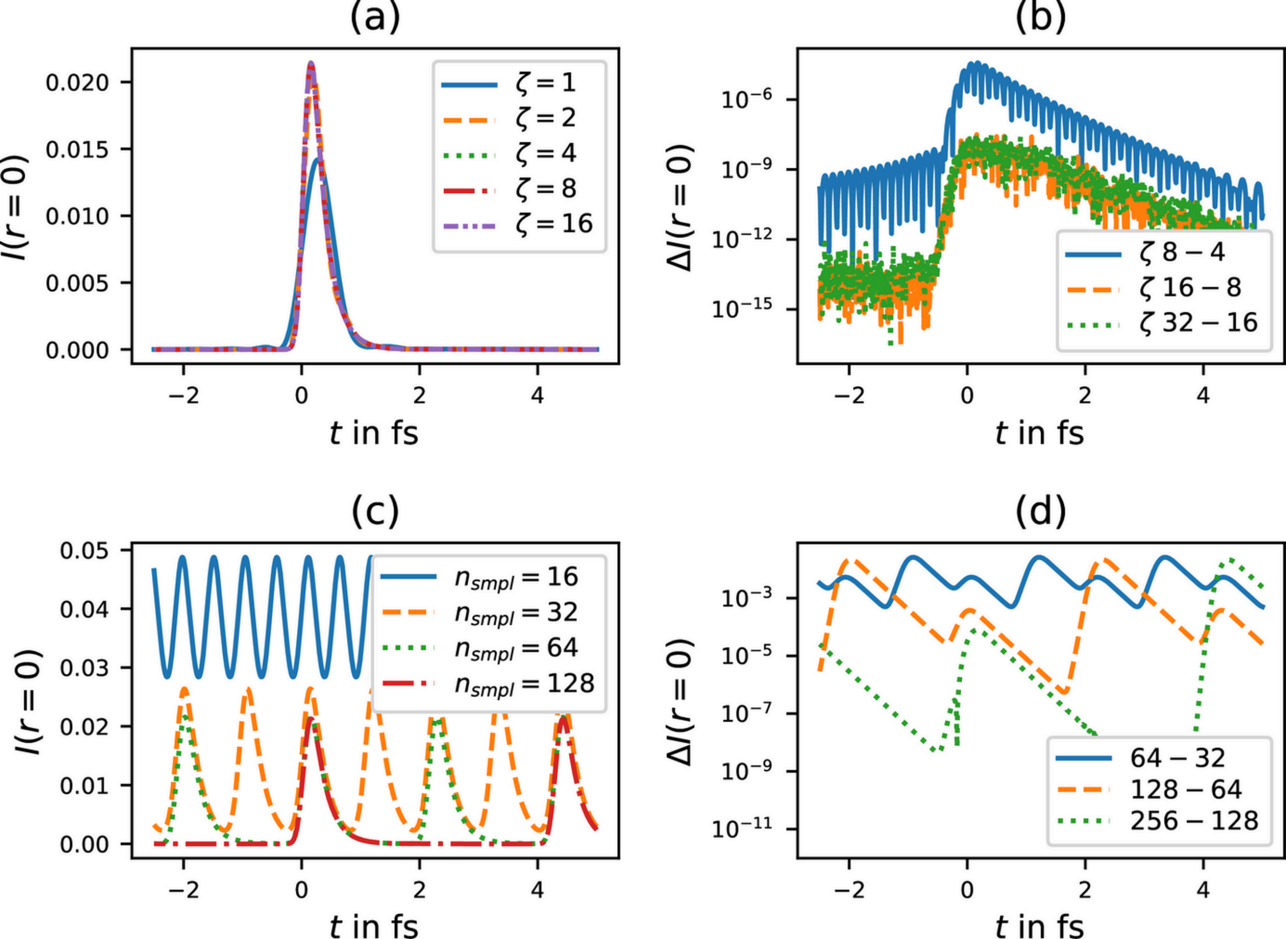
Numerical convergence test of equation (27). *I* = ψ^*^ψ evaluated at 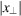 = *r* = 0, *z* = 1 m for *E*_0_ = 10 keV and *f* = 1 m. (*a*) Convergence when increasing the sampled frequency window. Evaluated with *n*_smpl_ = 512. (*b*) Difference when increasing ζ, *e.g.*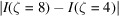; note the good convergence for ζ > 16. (*c*) Convergence when increasing the number of sampling points. Evaluated with ζ = 32. (*d*) Difference when increasing *n*_smpl_, *e.g.* |*I*(*n*_smpl_ = 64) − *I*(*n*_smpl_ = 32)|.

**Figure 12:**
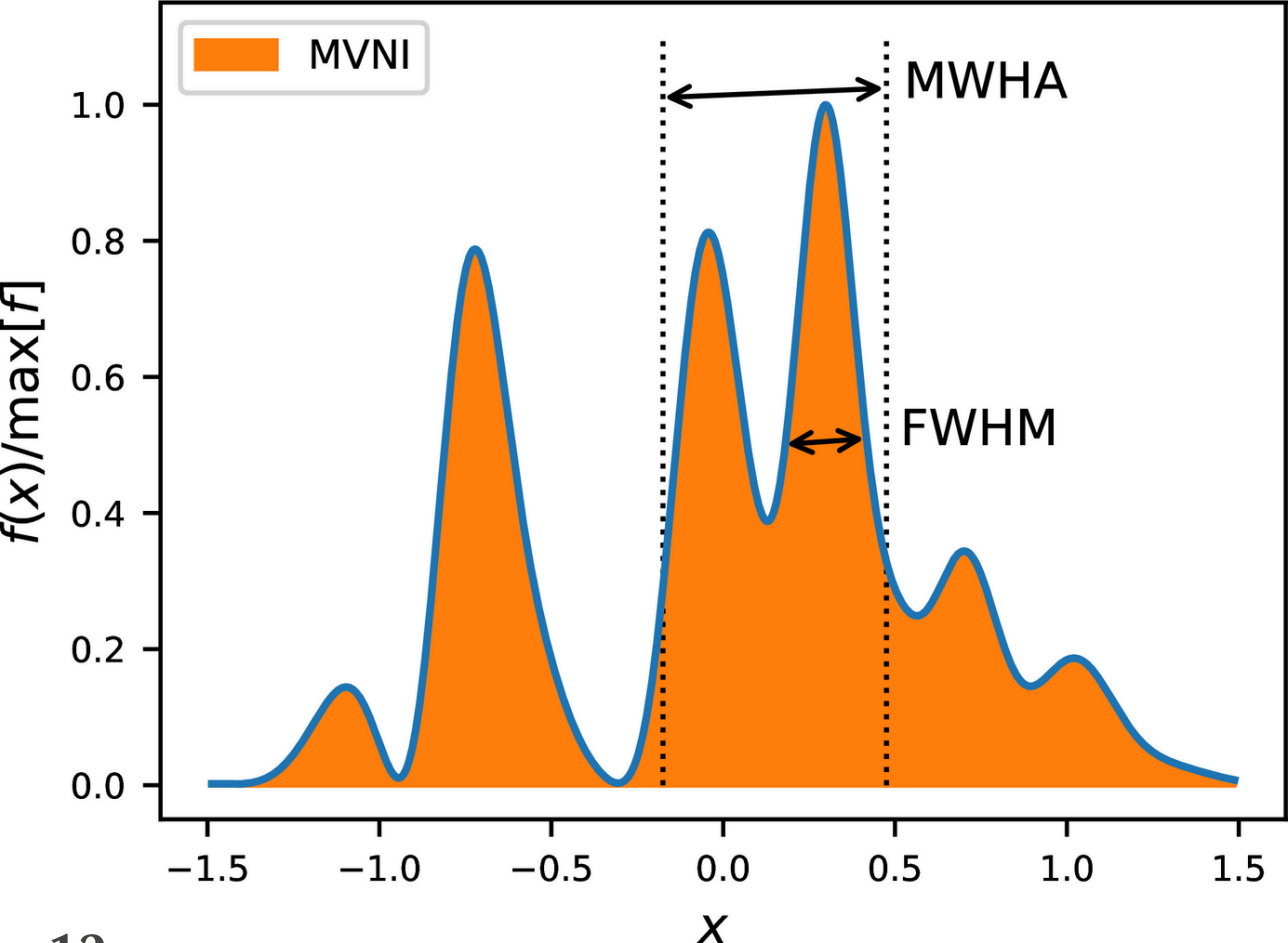
Example for the pulse width estimation of a typical 1 fs SASE pulse. Full width at half-maximum FWHM[*f*(*x*)] = 0.18, minimum width of half area MWHA[*f*(*x*)] = 0.65 and maximum value normalized integral MVNI[*f*(*x*)] = 0.835.

**Table 1:** Pulse lengths of the example pulses from Fig. 8 ▸ and the averages over the whole set Here we present two different averages, one before applying the ‘Width’ functional ({FWHM[〈*I*(*t*)〉], MWHA[〈*I*(*t*)〉], MVNI[〈*I*(*t*)〉]}) – and after applying the ‘Width’ functional to each sample (〈Width[*I*(*t*)]〉).

	Example 1	Example 2	Width[〈*I*(*t*)〉]	〈Width[*I*(*t*)]〉
FWHM initial	0.150 fs	0.327 fs	1.001 fs	0.315 ± 0.120 fs
FWHM focus	0.608 fs	0.454 fs	1.170 fs	0.632 ± 0.232 fs
FWHM focus/initial	4.061	1.387	1.169	2.198 ± 1.072
MWHA initial	0.392 fs	0.211 fs	0.575 fs	0.431 ± 0.161 fs
MWHA focus	0.516 fs	0.340 fs	0.670 fs	0.567 ± 0.144 fs
MWHA focus/initial	1.317	1.610	1.166	1.387 ± 0.235
MVNI initial	0.434 fs	0.390 fs	1.068 fs	0.570 ± 0.146 fs
MVNI focus	0.882 fs	0.593 fs	1.246 fs	0.876 ± 0.184 fs
MVNI focus/initial	2.035	1.520	1.167	1.565 ± 0.173

## Footnotes

1 Note that we define ‘intensity’ as  := , which deviates from the usual definition where intensity is defined as a time average 〈ψ^*^ψ〉_*t*_.

2 The parabola factor *a* is defined via the parabola function *f*(*x*) = *ax*^2^.

3 The Heaviside step function is defined as  := {0 if *t* < 0; 1/2 if *t* = 0; 1 if *t* > 0}.

4 Usually the Fresnel number is defined as *F*_*r*_ := *d*^2^/(*f*λ); however, we call *F*_*r*_ Fresnel number within this work for simplicity/convenience, since it is quite similar and we assume monochromaticity for our evaluations anyways, so λ is just a constant factor.

5 It is important to strictly distinguish between the ‘duration of the averaged pulse’ and the ‘averaged pulse duration’ within this section.

## Appendix A Propagation in Fresnel theory

For evaluating equation (7) it is actually possible to evaluate either the integral over ω or over  and obtain a closed form. However, we did not manage to evaluate both integrals successively and still obtain a closed form. A closed-form solution is likely not possible. Therefore we decided to evaluate the spatial integral analytically and obtain

With this, equation (7) becomes

Since there is no closed form solution for this integral,^6^ it needs to be evaluated numerically, which we discuss in the following.

### A1. Numerical propagation of Gaussian pulses

A strategy to numerically evaluate the integral in equation (24) is to sample the frequency ω around the central frequency ω_0_. This is justified since the term  suppresses contributions for ω varying substantially from ω_0_. The sampling approach can also be understood as a numerical Fourier transform. To determine the sampling window (as a function of σ_*t*_) we take a look at the power spectrum

We choose to express the sampling-window size in terms of σ_*E*_ multiplied by the window-size factor ζ_smpl_,

The integral in equation (24) can now be approximated as a sum,

The accuracy of this approximation is determined by the size of the sampling window (represented by ±σ_E_ζ_smpl_) and the number of sample points (given by 2*n*_smpl_ + 1).

To evaluate the convergence we only consider the intensity *I* = ψψ^*^. In Fig. 11 ▸ the numerical stability is demonstrated for different ζ_smpl_ and *n*_smpl_. For the evaluations within this paper, ζ_smpl_ ≥ 16 and *n*_smpl_ ≥ 512 were used to ensure a sufficient sampling.

## Appendix B Derivation of the effective Fresnel number in the ray-tracing model

To derive the effective Fresnel number , we start with equation (15) and Taylor expand the exponent around Δ*t* = 0 up to linear order

We extract the effective Fresnel number by solving

To validate this result, we Taylor expand the factor outside the exponential function, truncate it at constant order and identify ,

## Appendix C About the width of a curve

Even though well established, the FWHM turns out to be an insufficient metric when it comes to curves that consist of multiple spikes (as it is the case for SASE pulses). This is illustrated in Fig. 12 ▸, where the FWHM is defined as the distance between the two closest *x*-values left and right from the function maximum, where the function evaluates to half of the maximal value. An alternative definition would be to look for the most distant two points left and right from the maximum, but that definition would lead to a very similar problem.

To address this issue, we present two alternative measures to estimate the width of a curve. All presented methods require that the investigated curve *f*(*x*) is positive *f*(*x*) ≥ 0 ∀*x* and lives in the L^1^ Banach-space . All width metrics are represented as functionals, *e.g.* FWHM[*f*(*x*)].

### c1. Minimum width of half area

The minimum width of half area (MWHA) is defined as the minimum distance between two points (*x*_1_ and *x*_2_) where the integral is half of the integral over the entire function,

The MWHA is a good measure for the compactness of the function and might be useful when radiation damage is investigated and therefore energy deposition within a certain time is a crucial measure (*e.g.* Nass *et al.*, 2015 ▸). The dis­advantage is that the calculation of MWHA is computationally expensive.

### c2. Maximum value normalized integral

The maximum value normalized integral (MVNI) is defined as the integral of the function *f* after normalization by its maximal value,

The MVNI for a rectangular function would therefore be exactly the width of that function. However, when *f* consists of two separated peaks, the MVNI is insensitive to the distance of these peaks. Therefore the MVNI is a good measure of how concentrated the function is but neglecting distances for individual peaks. The MVNI is a useful measure when calculating speckle contrasts, *e.g.* in SAXS experiments (Yun *et al.*, 2019 ▸), or the visibility factor in IDI experiments (Trost *et al.*, 2023*b* ▸).
